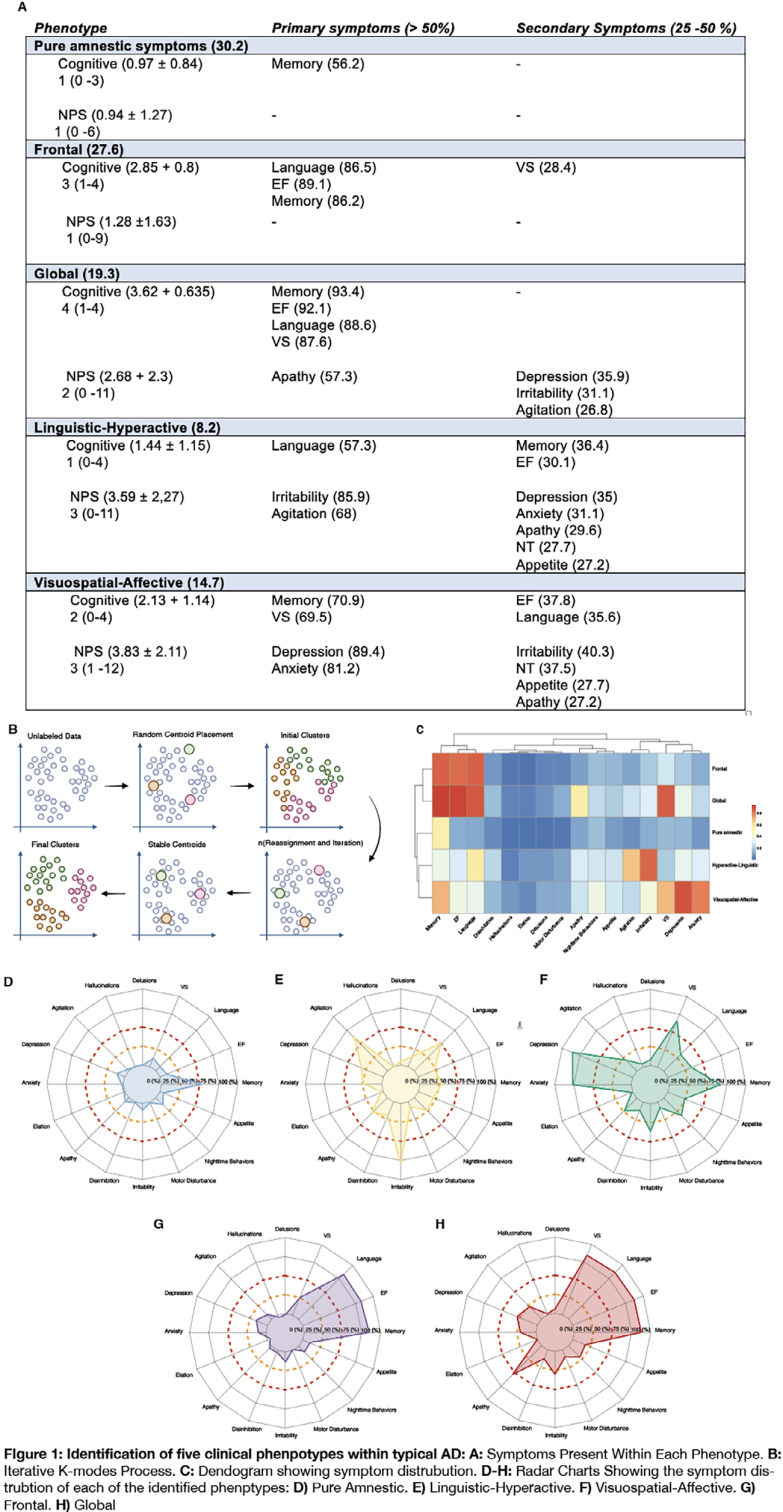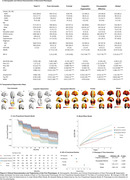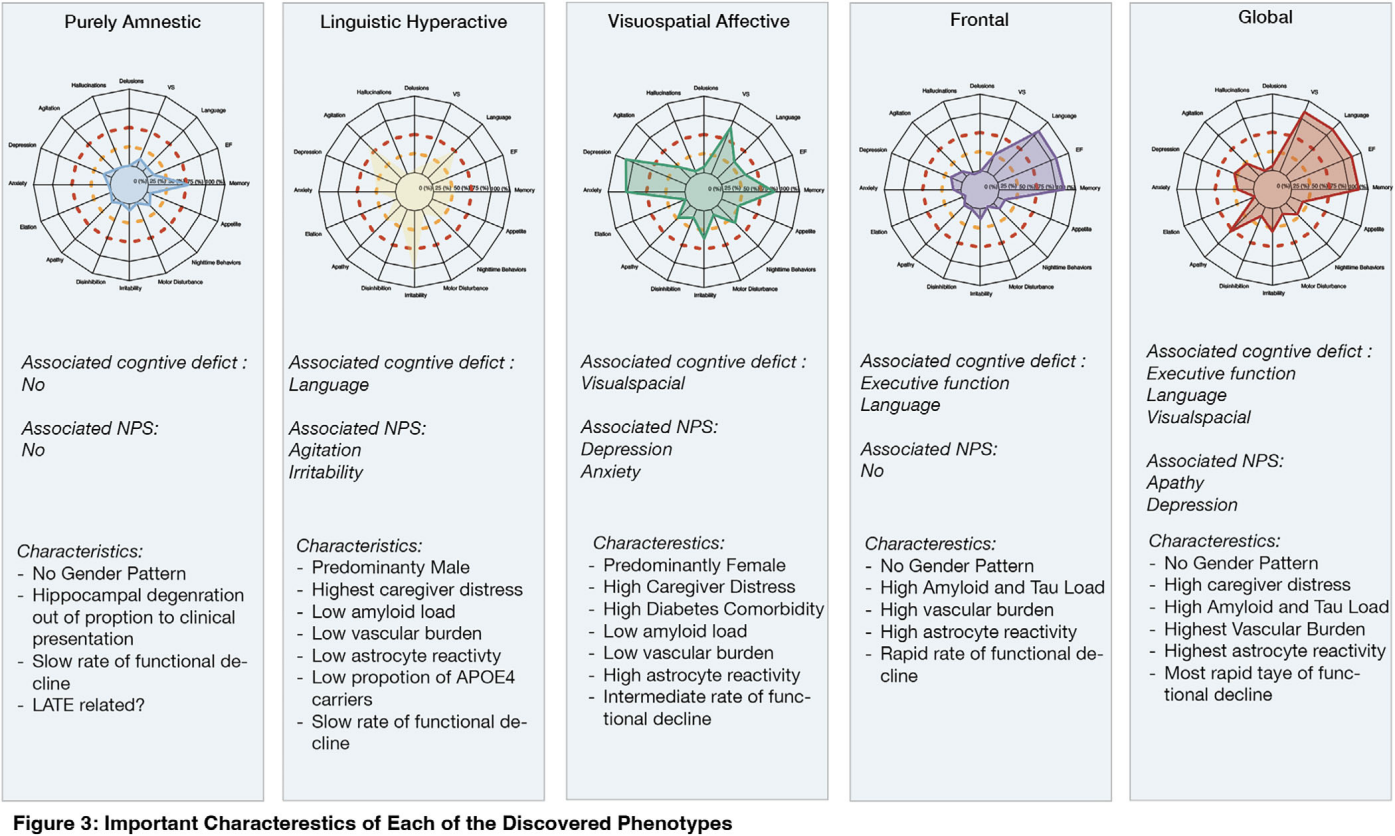# Robust Symptomatic Subtypes of Typical Alzheimer’s Disease Characterized by Differential Neuropathological Patterns and Functional Decline

**DOI:** 10.1002/alz.091322

**Published:** 2025-01-03

**Authors:** Hussein Zalzale, Pamela C.L. Ferreira, Peter Lemaire, Marina Scop Madeiros, Carolina Soares, Bruna Bellaver, Guilherme Bauer‐Negrini, Firoza Z Lussier, Guilherme Povala, João Pedro Ferrari‐Souza, Sarah Abbas, Cristiano Schaffer Aguzzoli, Matheus Scarpatto Rodrigues, Francieli Rohden, Livia Amaral, Douglas Teixeira Leffa, Markley Oliveira, Dana Tudorascu, Cécile Tissot, Nesrine Rahmouni, Nicholas J. Ashton, Kaj Blennow, Chang Hyung Hong, Sang Joon Son, Pedro Rosa‐Neto, Ann D Cohen, Oscar L. Lopez, Victor L Villemagne, Tharick A. Pascoal

**Affiliations:** ^1^ University of Pittsburgh, Pittsburgh, PA USA; ^2^ Universidade Federal do Rio Grande do Sul, Porto Alegre, Rio Grande do Sul Brazil; ^3^ Brain Institute of Rio Grande do Sul, PUCRS, Porto Alegre, RS Brazil; ^4^ McGill University, Montreal, QC Canada; ^5^ King’s College London, Institute of Psychiatry, Psychology & Neuroscience, Maurice Wohl Clinical Neuroscience Institute, London United Kingdom; ^6^ University of Gothenburg, Gothenburg Sweden; ^7^ Ajou University School of Medicine, Suwon Korea, Republic of (South); ^8^ Ajou University Hospital, Suwon Korea, Republic of (South)

## Abstract

**Background:**

Alzheimer’s disease (AD) is classically viewed as a predominantly amnestic syndrome, with other cognitive and neuropsychiatric symptoms (NPS) being non‐integral associations. Emerging Evidence suggests that within typical AD, these symptoms are core features from the onset.

**Methods:**

We employed K‐modes clustering on 2483 cognitively impaired (CI) individuals (CDR ≥ 0.5), excluding participants diagnosed with atypical AD, non‐amnestic MCI, or CI due to non‐AD dementias from five cohorts: TRIAD, ADNI, BICWALZS, OASIS‐III, and Pittsburgh. Cluster‐specific clinical and pathological profiles were established through comparison with 2670 cognitively unimpaired (CU) participants across plasma biomarkers (Ptau‐181, Ptau‐217, GFAP, Nfl, AB42/40 ratio) and neuroimaging (amyloid and tau PET, white matter hyperintensity, MRI‐derived degeneration maps). The rate of functional decline, modeled by increase in CDR‐SB, was assessed using a Cox‐proportional hazards model and a linear mixed‐effect model.

**Results:**

We identified five distinct clinical phenotypes within typical AD: ‘Pure Amnestic’ (30.2%), ‘Linguistic‐Hyperactive’ (14.7%), ‘Visuospatial‐Affective’ (14.7%), ‘Frontal’ (27.6%), and ‘Global’ (19.3%) (**Figure 1, Figure 3**). These phenotypes demonstrated consistency regardless of amyloid status or cohort. Each phenotype exhibited a unique neuropathological signature and a distinct pattern of neurodegeneration (**Figure 2**). A critical aspect of our findings is the differential rate of functional decline across these phenotypes. The ‘Pure Amnestic’ group showed the slowest decline, followed by ‘Linguistic‐Hyperactive’ (HR = 1.39; B = 0.2), ‘Visuospatial‐Affective’ (HR = 2.13; B = 1), ‘Frontal’ (HR = 2.23; B = 1.53), and the ‘Global’ phenotype showing the fastest decline (HR = 3.52; B = 2.66) (**Figure 2, Figure 3**).

**Conclusion:**

Our results challenge the concept of “typical” AD by uncovering five robust clinical phenotypes with divergent neuropathological profiles and clinical characteristics. These results mark an important advancement toward personalized medicine in the landscape of emerging AD treatments.